# *Cooltools*: Enabling high-resolution Hi-C analysis in Python

**DOI:** 10.1371/journal.pcbi.1012067

**Published:** 2024-05-06

**Authors:** Open2C, Nezar Abdennur, Sameer Abraham, Geoffrey Fudenberg, Ilya M. Flyamer, Aleksandra A. Galitsyna, Anton Goloborodko, Maxim Imakaev, Betul A. Oksuz, Sergey V. Venev, Yao Xiao

**Affiliations:** 1 https://open2c.github.io/; 2 Department of Genomics and Computational Biology, University of Massachusetts Chan Medical School, Worcester, Massachusetts, United States of America; 3 Department of Systems Biology, University of Massachusetts Chan Medical School, Worcester, Massachusetts, United States of America; 4 Institute for Medical Engineering and Sciences, Massachusetts Institute of Technology (MIT), Cambridge, Massachusetts, United States of America; 5 Department of Quantitative and Computational Biology, University of Southern California, Los Angeles, California, United States of America; 6 Friedrich Miescher Institute for Biomedical Research, Basel, Switzerland; 7 Institute of Molecular Biotechnology of the Austrian Academy of Sciences (IMBA), Vienna BioCenter (VBC), Vienna, Austria; Simon Fraser University, CANADA

## Abstract

Chromosome conformation capture (3C) technologies reveal the incredible complexity of genome organization. Maps of increasing size, depth, and resolution are now used to probe genome architecture across cell states, types, and organisms. Larger datasets add challenges at each step of computational analysis, from storage and memory constraints to researchers’ time; however, analysis tools that meet these increased resource demands have not kept pace. Furthermore, existing tools offer limited support for customizing analysis for specific use cases or new biology. Here we introduce *cooltools* (https://github.com/open2c/cooltools), a suite of computational tools that enables flexible, scalable, and reproducible analysis of high-resolution contact frequency data. *Cooltools* leverages the widely-adopted cooler format which handles storage and access for high-resolution datasets. *Cooltools* provides a paired command line interface (CLI) and Python application programming interface (API), which respectively facilitate workflows on high-performance computing clusters and in interactive analysis environments. In short, *cooltools* enables the effective use of the latest and largest genome folding datasets.

## Author summary

Chromosome conformation capture (3C) experiments, including Hi-C, measure the 3D organization of chromosomes inside cells. As 3C datasets grow larger and higher-resolution, analyzing them poses computational challenges. Our open-source Python package cooltools meets these challenges. Cooltools integrates smoothly with cooler, a software library for storing and accessing very large Hi-C datasets. Cooltools enables users to extract key features seen in 3C maps, including: the decay of contact frequency with genomic distance, plaid patterns of active and inactive compartments, domains, and dots. Cooltools quantifies these folding features from raw contact data efficiently, handling chromosome-scale datasets too large to fit in memory. The command-line and Python interfaces make cooltools easy to integrate into pipelines or customized for new analyses. By overcoming data size and memory hurdles, cooltools allows researchers to harness 3C’s full potential for understanding principles of genome folding and function.

This is a *PLOS Computational Biology* Software paper.

## Introduction

Chromosome conformation capture technologies are powerful tools for experimentally characterizing higher-order genome organization [1]. Multiple labs and consortia, including the 4D Nucleome project (4DN) [2], the International Nucleome Consortium (INC), and ENCODE [3], are generating increasingly larger datasets to probe genome organization at ever higher resolutions. Larger datasets increase the challenges at each step of computational analysis, from storage to memory, to researchers’ time—at the highest resolutions, the data for even a single chromosome does not fit easily in memory.

Computational analyses of genome folding typically employ multiple approaches to quantify the variety of patterns in contact maps. These include: (i) enrichment of contacts within chromosomes, referred to as *chromosome territories*, (ii) decreasing contact frequency versus distance within chromosomes, or *P(s)*; (ii) plaid patterns of preferential contacts, referred to as *compartmentalization*, (iii) squares of enriched contact frequency, referred to as *topologically associated domains* (TADs), (iv) focal peaks of pairwise contact frequencies, referred to as *loops* or *dots*. To quantitatively describe these features, and assay how they change across conditions, maps are summarized in many ways. Summaries include 2D pairwise averages, 1D profiles, and lists of genomic positions. The variety of Hi-C features and summaries benefits from a set of modular tools, rather than end-to-end pipelines, for data analysis.

The features in genome folding maps vary substantially through cell states, across organisms, and between protocols. As C-data is obtained from a growing variety of contexts, computational analyses must co-evolve and expand as well. Because of this, computational researchers require APIs that can not only perform standard analyses but also enable the design of new analyses. This includes flexibility over parameters and thresholds, but also encompasses more dramatic extensions.

Python provides one of the most popular environments for flexible data analysis and software development. This is in part due to the rich ecosystem of *numpy [4]*, *scipy* [5], *scikit-learn* [6], and *pandas [7]*. For Hi-C analysis in Python, the widely-adopted *cooler [8]* format and library present an ideal foundation. *Cooler* handles storage and access for high-resolution datasets via a sparse data model. To fully leverage the advantages of *cooler*, however, requires a corresponding analysis framework in *Python*.

While there are existing computational suites for extracting features of genome folding via the CLI [9–13] or an R API [14], only FAN-C [13] and TADbit [11] have Python APIs (**Table 1**). However, FAN-C and TADbit neither take full advantage of the Python ecosystem nor provide consistent low-level access to the data, limiting their flexibility. We note the TADbit framework was developed before *cooler* was available, and hence without sparse storage considerations, and had an orthogonal primary focus on building 3D models.

**Table 1:** Overview of software suites for C-data analysis.

*Tool suite*	CLI	API	Storage
*cooltools*	Yes	Python	.cool
*FAN-C*	Yes	Python	.cool or.hic
*TADbit*	Yes	Python	.bam,.txt
*Genova*	No	R	.cool or.hic
*HiCExplorer*	Yes	No	.cool or.hic
*JuicerTools*	Yes	No	.hic
*HiC-Bench*	Yes	No	.tsv

Despite the multiple existing suites for extracting features of genome folding from Hi-C maps, only *cooltools* and *FAN-C* both have a Python API and accept input files with a standard binned Hi-C format. Currently, the standard binned formats used by the 4D Nucleome project [2] and others are: (i).cool, the hdf5-based storage format used by cooler [8], and (ii).hic, the custom binary format generated by Juicer [12].

**Fig 1:**
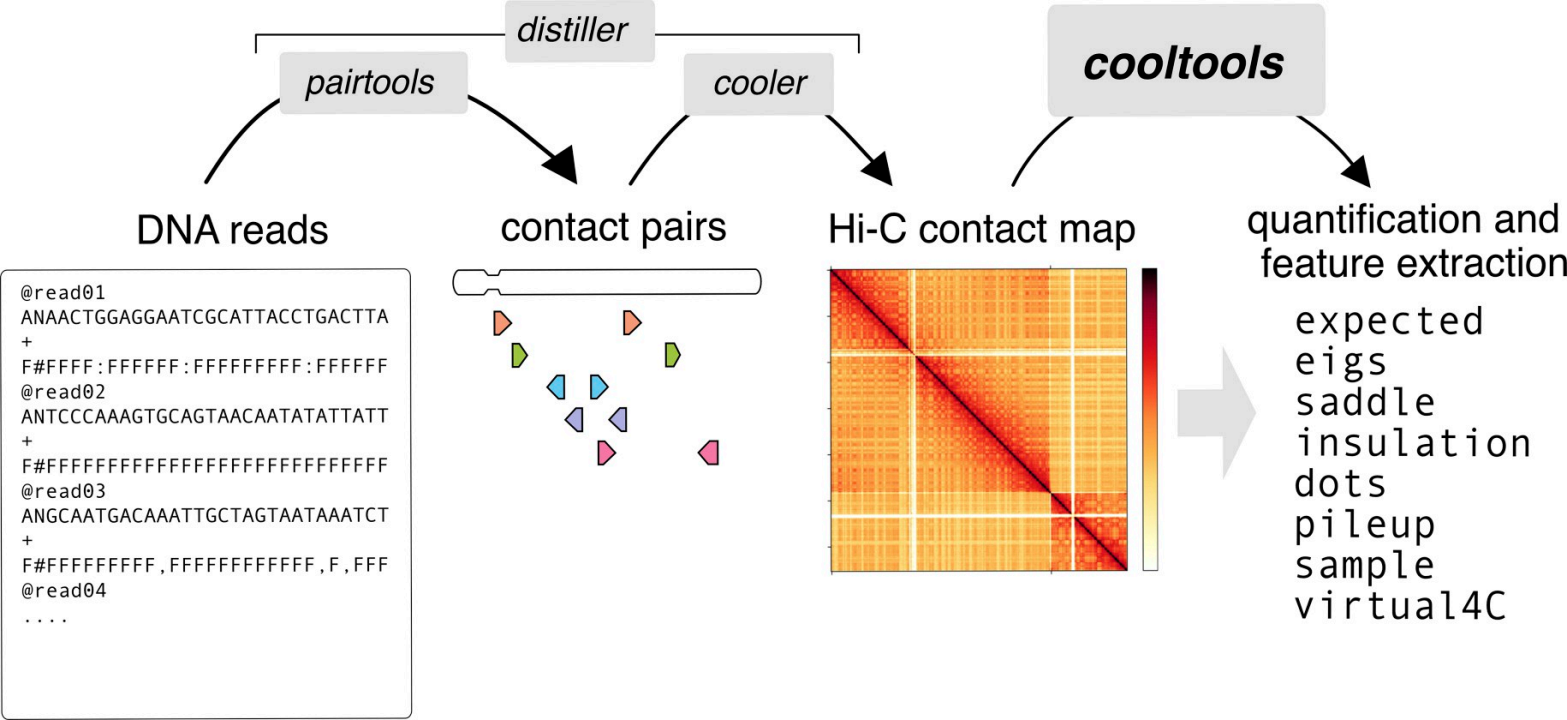
Overview of cooltools functionality. Open2C provides a modular ecosystem of software libraries for Hi-C analysis (highlighted with gray boxes). Pairtools [58] takes in paired-end sequence alignments and extracts contact pairs in the 4DN.pairs format. cooler [8] bins these contact pairs and stores the resulting sparse matrices in.cool and.mcool formats. The nextflow pipeline distiller [59] converts sequencing reads from FASTQ files directly into binned and normalized cooler files, integrating read alignment with pairtools and cooler. The library introduced in this paper, cooltools (in bold), provides methods to quantify and extract features from high-resolution contact maps stored by cooler.

Here we introduce *cooltools*, a suite of computational methods that enable the analysis of high-resolution genome folding data accessible using *cooler* (**Fig 1**). *Cooltools* features a Python API paired with command line tools. The methods in *cooltools* are modular and provide access to both high-level and low-level APIs. The high-level API enables users to reproducibly perform routine analysis. The low-level API allows for troubleshooting challenges posed by new organisms or cell states and developing new advanced analyses. *cooltools* is part of a research community and broader ecosystem of open-source chromosome data analysis tools, Open2C (https://open2c.github.io/). *cooltools* provides detailed introductions to key concepts in Hi-C-data analysis with interactive notebook documentation. Collectively, *cooltools* enables computational analysis and methods development to keep pace with the rapidly-increasing quantity of experimental data and rapidly-evolving understanding of genome organization.

### Design and implementation

The design of *cooltools* stems from its goal to enable flexible analyses of large quantities of high-resolution Hi-C data.

1. Provide a modular set of tools with a balance between comprehensiveness and flexibility. Modular tools can be used separately or combined in a custom order. The set of core tools extracts and quantifies the majority of common features in the Hi-C literature.
2. Separate processing from data analysis. *Cooltools* works with processed Hi-C data and is not concerned with mapping workflows.
3. Separate data analysis from plotting, with a focus on the former. For Hi-C data, plotting often has a greater range of user-specific needs than feature extraction and quantification.
4. Provide a paired Python API and CLI. CLI tools enable their use in computational pipelines and have a one-to-one correspondence with user-facing Python API functions. The user-facing API and CLI both have a standard format for arguments and take a cooler as their first input. For other types of genomic data besides Hi-C, the cooltools API accepts *pandas* dataframes, while the CLI reuses existing formats when possible: BED-like files for interval data and bedgraphs or bigwigs for genomic tracks.
5. Split the Python API into two layers: a high-level API for user-facing functions and a flexible low-level APIs. A modular, consistent, and well-documented internal Python API allows experienced users to customize existing analysis routines or create entirely new ones.
6. Enable analyses for custom genomes and non-model organisms. This includes user-defined subsets of regions from a reference genome, e.g. chromosomal arms. Leveraging its use of standard Python data structures, cooltools enables these analyses with *bioframe [15]*. A view specifies a unique 1D coordinate axis with an ordered set of non-overlapping genomic intervals, also referred to as *regions*.
7. Leverage indexed sparse storage. *Cooltools* is built directly on top of the *cooler* storage format and library, which allows it to operate on sparse matrices and/or out-of-core, either on raw counts or normalized contact matrices.
8. Allow parallel processing to take advantage of modern multi-core CPU architectures. Most operations are performed via iteration over genomic regions or chunks of non-zero pixels, enabling straightforward multiprocessing.
9. Leverage the Python data science stack to promote interoperability. *Cooltools* delegates to *numpy*, *scipy*, and *pandas* when possible and aims to follow the style of these APIs. To accelerate specific low-level computations, *cooltools* uses the just-in-time compiler *numba [16]*.
10. Return widely-used Python data structures, *pandas* dataframes, and *numpy* arrays, for user-facing outputs, and avoid the use of custom classes. This allows *cooltools* to operate on low-level and easily modifiable Python objects with an intuitive, consistent, and flexible interface.

*Cooltools* is open-source and structured to encourage future contributions. For experimental code, *cooltools* provides a sandbox to enable easy sharing, safe testing, and eventual adoption of novel analysis routines.

## Results

### Overview

The main *cooltools* modules were initially developed with mammalian interphase Hi-C maps in mind, but the flexible implementation of *cooltools* has made it useful for analysis of data from different organisms (e.g., *Drosophila* [17,18], yeast [19,20], nematodes [21], chicken [22]) and cellular states (mitosis [22,23], meiosis [20,24]). We explain *cooltools* in tripartite chapters for each module below, describing the relevant features, the implementation, and analyses where the flexible API has proven useful. Conceptually, *cooltools* modules can be grouped in two main ways. First, modules can be grouped by whether they perform feature extraction (compartments, insulation, dots) versus feature quantification (expected, saddles, pileups). Alternatively, modules can be grouped by whether they make use of chromosome-wide information (expected, compartments, saddles) or more local information (insulation, dots, pileups). The design choice to separate analysis from plotting facilitated the creation of interactive notebook documentation (https://cooltools.readthedocs.io/en/latest/notebooks). These notebooks also provide flexible starting points for custom visualization and analysis. To highlight the practical application of *cooltools*, the figures throughout this manuscript are all generated using the same HFF micro-C dataset from Krietenstein et al., [25] stored as an.mcool file, which provides a convenient access to sparse matrices binned at multiple resolutions, and the code to generate all figures is additionally available for download as interactive notebooks (https://github.com/open2c/open2c_vignettes/tree/main/cooltools_manuscript).

### Contacts-vs-distance & expected

Contact frequency decreases rapidly with genomic separation within chromosomes, which arises because chromosomes are long polymers [26] This feature is interchangeably referred to as the: (*i*) *expected* because locus-specific features increase or decrease contact frequency relative to this major trend, *(ii) scaling* which is borrowed from the polymer physics literature, (*iii*) *P(s)*, or contact probability, *P*, as a function of genomic separation, *s*. The shape of *P(s)* reflects folding patterns at different length scales, from those of adjacent nucleosomes to full chromosomes [26,27], and can be quantified with its derivative. *P(s)* varies through the cell cycle, across cell types, and is modified by structural maintenance of chromosomes complexes (SMCs) in interphase, mitosis, and meiosis (review [28]).

**Fig 2:**
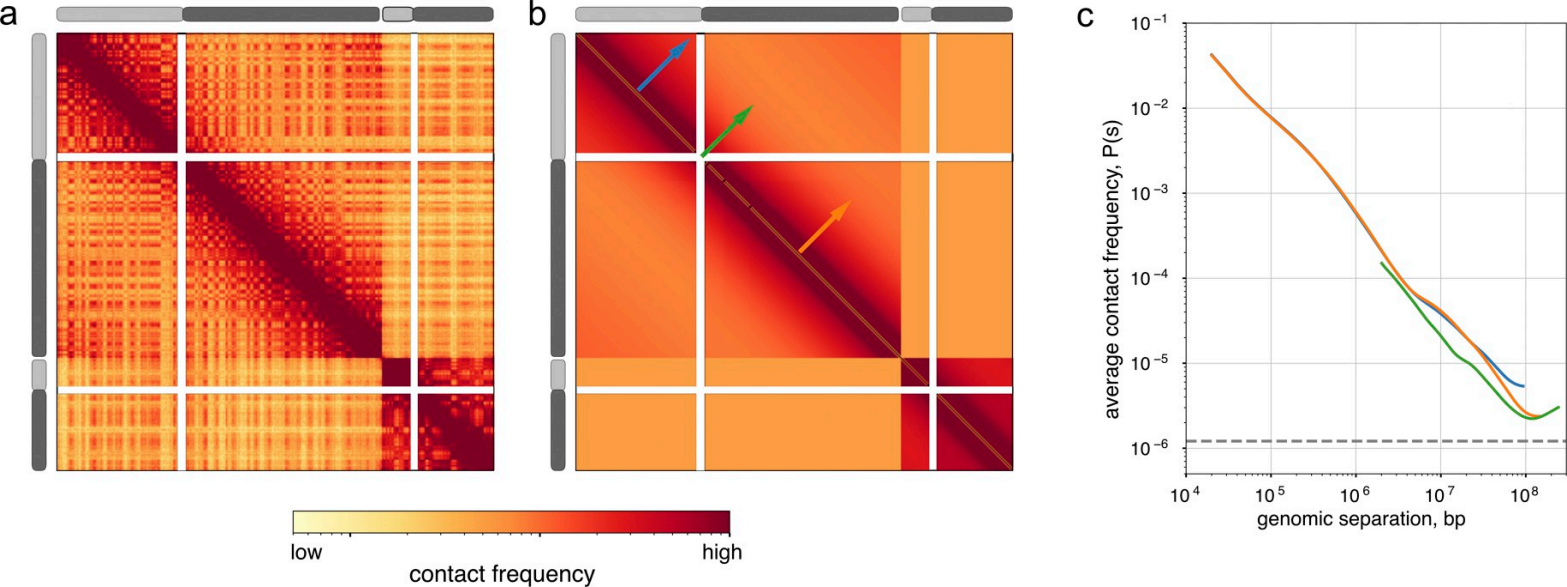
Expected and contact frequency versus distance. ***a*.** Observed contact map for HFF Micro-C for chr2 and chr17 at 1Mb. Chromosomal arms p and q are depicted as light and dark grey rectangles respectively. Note the wide unmappable centromeric regions (white rows and columns) between chromosomal arms. Accounting for these regions is a key aspect of calculating an expected map. ***b*.** Expected map for three classes of regions: intra-chromosomal intra-arm, intra-chromosomal inter-arm, and inter-chromosomal. Regions for expected are specified using genomic views, where individual regions are chromosomal arms. Note that intra-chromosomal expected has a strongly decreasing contact frequency with genomic distance, whereas inter-chromosomal expected appears flat. ***c*.** Average contact frequency versus genomic separation, or *P(s)*, for intra-arm interactions (blue, orange) and for inter-arm interactions (green), calculated from contact maps at 10kb. *P(s)* curves are matched by region and color with arrows on the middle heatmap.

To quantify *P(s)* at sub-kilobase resolution (**Fig 2**), *cooltools* avoids conversion from sparse-to-dense matrices. Instead, the calculation is done by iterating over chunks of pixels stored in coolers, assigning pixels to regions, and aggregating at each distance. Avoiding conversion to dense also requires keeping track of filtered-out bins and a non-trivial accounting of the number of intersecting filtered bin pairs per-region per-distance. By allowing users to specify a correction weight, *cooltools* also enables *P(s)* calculation on raw data. To reduce variation at large genomic separations due to increasing sparsity of the data, *cooltools* returns *P(s)* curves smoothed in log-space using a *numba*-accelerated approach. This also allows *cooltools* to more robustly compute the derivative of *P(s)* and observed-over-expected maps. To quantify *P(s)* for arbitrary sets of regions, users can specify a view. The resulting curves for each region are then optionally averaged together. To enable interoperability with downstream analyses that require accounting for *P(s)*, *cooltools* defines an expected format with specifications and checks. This includes both pairs of regions within chromosomes (*cis* expected) or pairs of regions on different chromosomes (*trans* expected).

The flexible approach for computing *P(s)* used in *cooltools* has proven useful in multiple situations. For example, the ability to specify regions with a view has been used to calculate both within-arm *P(s)* and within-compartment *P(s)*. Within-arm *P(s)* was particularly useful in mitosis, as P(s) is the main point of comparison with models of large-scale chromosome organization and is altered greatly across centromeric regions [22]. Within-compartment P(s) [29] revealed that extrusion occurs in compartment B as well as in A.

### Compartmentalization

A second prominent signal in mammalian interphase contact maps is plaid patterns that appear both within and between chromosomes, termed chromosome compartments. These plaid patterns reflect tendencies of chromosome regions to make more frequent contacts with regions of the same type: active regions have increased contact frequency with other active regions, and inactive regions tend to contact other inactive regions more frequently. The strength of compartmentalization varies through the cell cycle, across cell types, and after the degradation of components of the cohesin complex (for review [28]).

**Fig 3:**
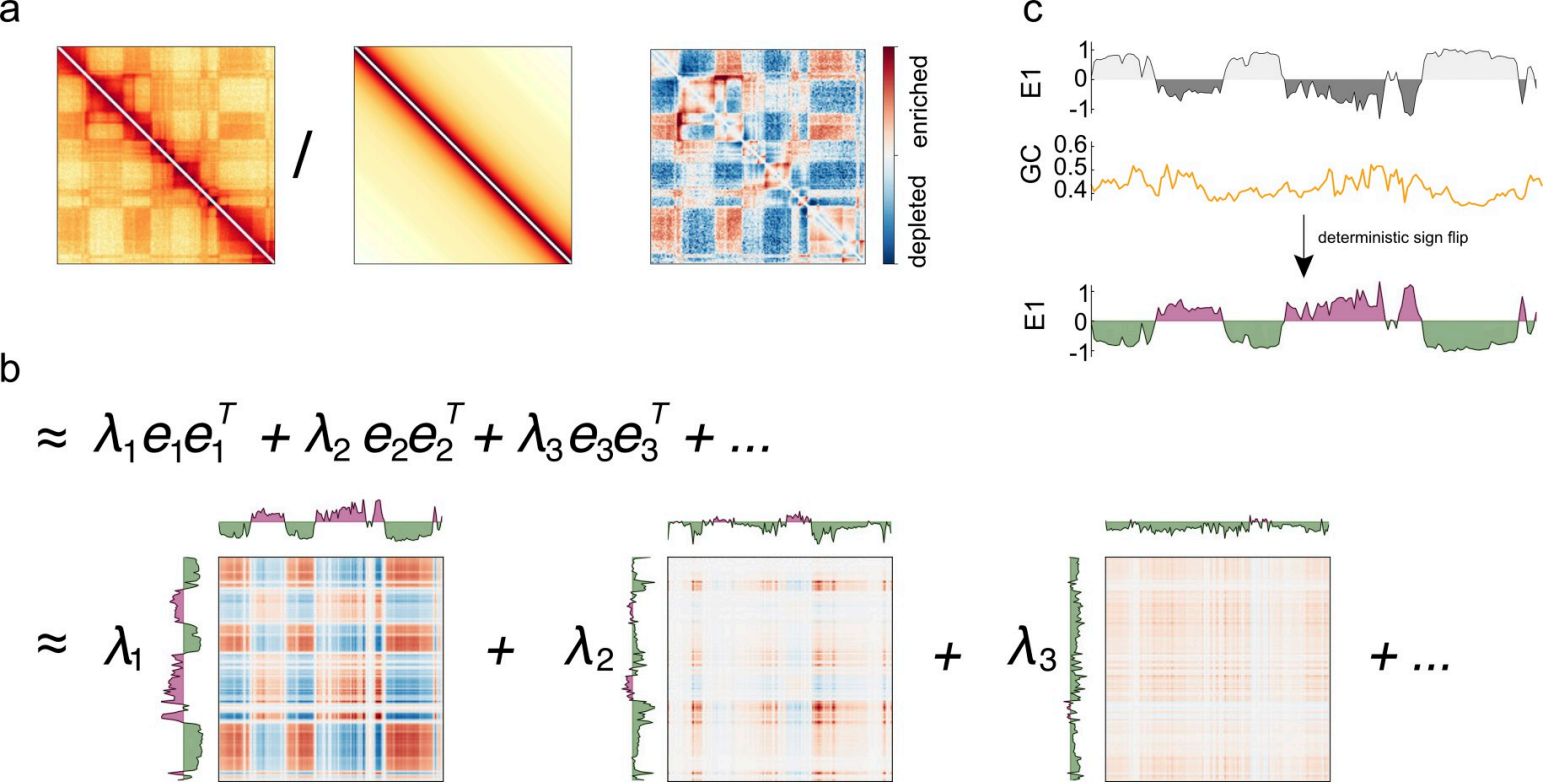
Compartments and eigenvectors. **a.** To obtain cis compartments profiles, observed maps are first divided by expected. **b.** Observed/expected maps are decomposed into a sum of eigenvectors and associated eigenvalues. **c.** Illustration of eigenvector phasing. In mammalian Hi-C maps, the first eigenvector typically, but not always, corresponds to the compartment signal. Since eigenvectors are determined only up to a sign, their orientations are random. To obtain consistent results, the final cis compartment profile (right) is obtained as the eigenvector most correlated with a phasing track (here, GC content), and oriented to have a positive correlation.

*Cooltools* calculates compartmentalization profiles using matrix eigenvector decomposition (**Fig 3**). For *cis* profiles, *cooltools* first adjusts the contact matrix for distance dependence before decomposition. For *trans* profiles, *cooltools* implements the ICE approach which masks *cis* portions of the map with randomly sampled *trans* data. Since eigenvectors are determined up to a sign and compartmentalization patterns are sometimes better captured by second or third eigenvectors, calculated vectors often need to be oriented and sorted according to their correlation with known markers of activity (e.g., GC content, density of genes). *Cooltools* allows users to request an arbitrary number of eigenvectors and can sort and orient eigenvectors according to a provided “phasing track”, most commonly the GC content per genomic bin.

The flexible implementation makes it useful for downstream analysis. For example, [29] modifies *cooltools* to extract 50+ eigenvectors, which are then used to find multiple classes of compartments. In *Drosophila*, GC content did not always reliably orient eigenvectors, but the flexible interface allowed gene density to be used instead [17].

### Pairwise class-averaging & saddles

Contact frequency preferences between pairs of genomic loci can depend on multiple properties, including chromatin state and position along a chromosomal arm [30]. Pairwise summaries, obtained by averaging over pairs of regions assigned to a set of classes, are a straightforward way to quantify these preferences. Pairwise summaries are commonly visualized as a 2D heatmap. For genome folding, these are most frequently used to quantify the strength of compartmentalization after assigning classes from the extracted compartment profile. The pattern of preferences revealed by this averaging (where contacts are disfavored between active and inactive chromatin) led to these summaries being referred to as “*saddle plots*” [31]. Pairwise summaries have also been used to quantify GC content and fragment length biases [32].

**Fig 4:**
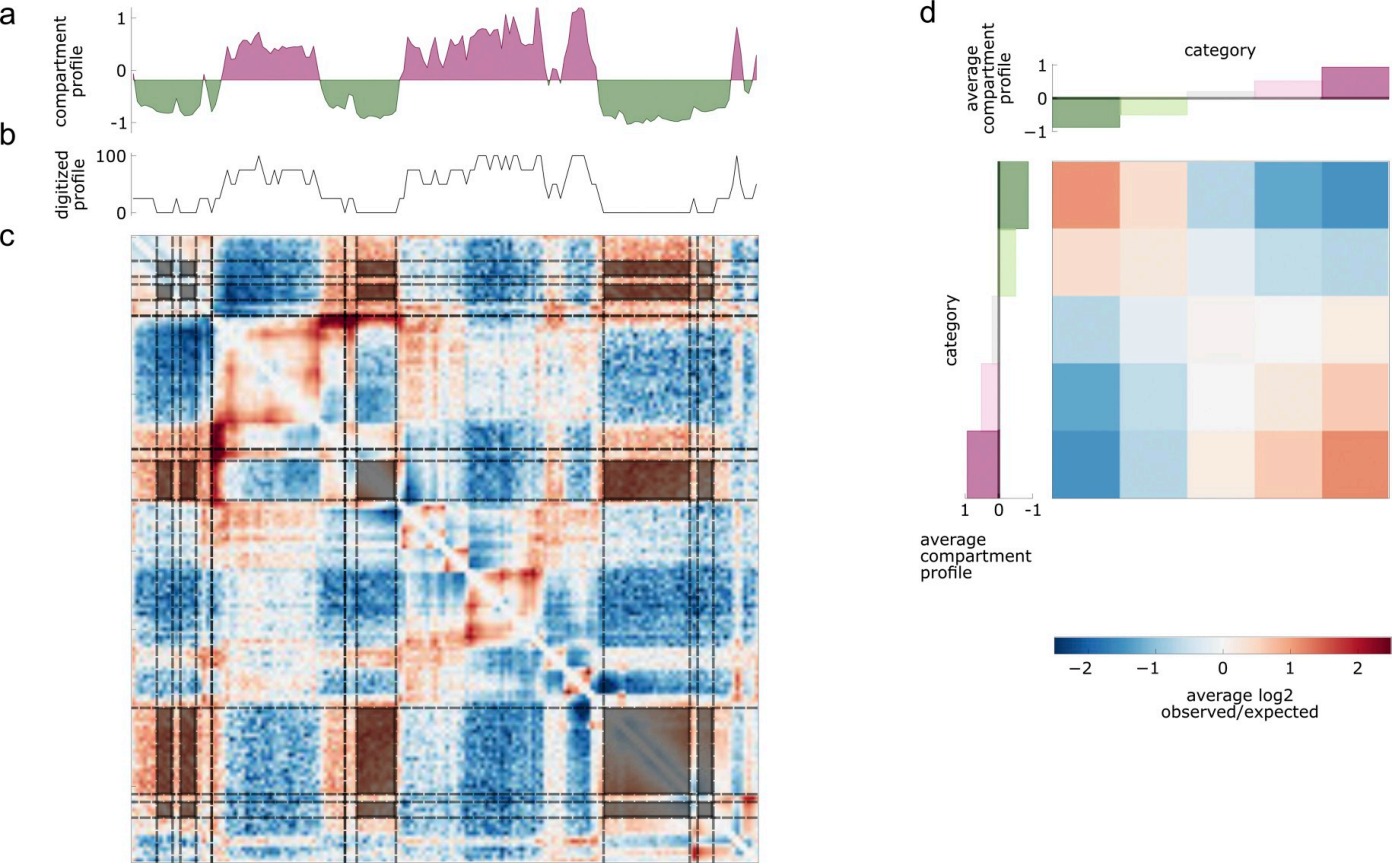
Pairwise class averaging and saddle plots. ***a*.** Compartment profile, where more negative values are B regions, and positive values A for a 15Mb region of chr2. ***b***. Digitized compartment profile, quantized into 5 classes by percentile. The lowest is highlighted as a thicker line. ***c*.** Observed/expected map with pairs of B regions highlighted. ***d*.** Saddle-plot for the 5 digitized classes, highlighted regions in the observed/expected map contribute to the top left pixel boxed in grey.

To quantify these preferences with *cooltools saddle* (**Fig 4**), the approach is to: (i) group bins with similar properties into *classes*, (ii) average over all map pixels specified by the bins in each pair of classes (i.e. for each pair of classes *p*, *q*, summarize the collection of pixels {*A*_*ij*_ | *i* in *p*, *j* in *q*}). Bins can be assigned to classes either by discrete properties, or by discretizing a quantitative signal. *cooltools* operates on observed-over-expected contact frequencies, either based on raw counts or balanced data, and can be restricted to either *cis* or *trans* portions of the genome wide map.

The flexibility of *cooltools* saddle enables 2D summaries to be used in many ways. This includes considering the average pairwise preferences over a reduced set of four histone modification states [29]. Saddle plots have also been used to quantify contact preferences relative to the nuclear lamina and speckles [33], as well as differences between experimental protocols [34]. Finally, since extraction of compartment profiles is decoupled from quantification, *cooltools* can be used to quantify how compartmental preferences shift with respect to a reference map, either through the cell cycle [23] or in single cells [35].

### Insulation and boundaries

At small genomic distances (~2 Mb and shorter), mammalian interphase contact maps display square-like features along the diagonal. These 2D features, also referred to as domains, or TADs, are often summarized with 1D profiles of *insulation*. Insulation profiles quantify the frequency of contacts across each genomic bin averaged over a sliding diamond-shaped window. Sites with lower scores, reflecting reduced contact frequencies between upstream and downstream loci, are often referred to as insulating *boundaries* and coincide with the edges of domains. Insulation scores are popular for analysis due to their simplicity and relative robustness to Hi-C sequencing depth [36]. Obtaining the positions of boundaries along the genome has in turn helped uncover relationships between genome folding and key genome-organizing proteins like CTCF and cohesin.

**Fig 5:**
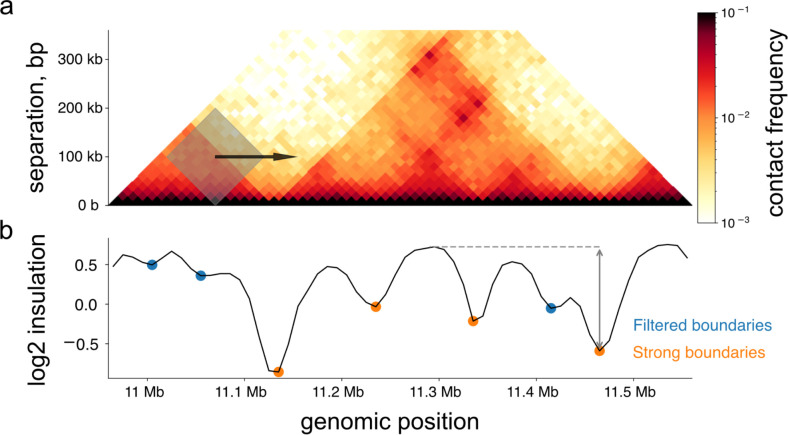
Insulation and boundaries. ***a*.** Diamond insulation is calculated as the sum in a sliding window (gray) across the genome, shown here for HFF MicroC data in a region of chr2 at 10kb resolution (chr2:10900000–11650000). ***b*.** The resulting insulation profile is shown in black. Local minima are indicated with dots. Positions of strong boundaries shown as orange dots, and filtered weak boundaries as blue dots. Two-sided gray arrow shows the boundary strength of the strong boundary at chr2:1146000–1147000, calculated relative to the maximum insulation achieved before a more prominent minima in either genomic direction. Here, strength is relative to the prominent minima at chr2:11130000–11140000, and maximum insulation is indicated with a dashed gray line.

To enable calculation of insulation profiles for high resolution data (**Fig 5**), *cooltools* iterates and aggregates over the sparse *cooler* pixel table. The size of the sliding window is chosen by the user. *cooltools* detects insulating boundaries as local minima of the insulation profile and quantifies their strength as their topographic prominence. Random variations in insulation along the genome lead to many local minima with very small prominences that are not desired in downstream analysis. To select strong boundaries from this set of minima, cooltools relies on Li and Otsu automated thresholding criteria borrowed from the image processing field (from scikit-image [37]).

*Cooltools* can calculate insulation profiles and boundaries at extremely high resolution (100 and 200 bp resolution [25,38]). The flexible window size has also been used to analyze Micro-C boundaries at multiple levels [38]. Finally, the flexibility of *cooltools* enabled its use to calculate insulation even with assays modified to detect sister chromatids [39,40].

### Dots

Punctate pairwise peaks of contact frequency are another prevalent feature of mammalian interphase contact maps. These features are also referred to as ‘dots’ or ‘loops’ in the literature, and can appear either in isolation or as parts of grids and at the corners of domains. In mammalian interphase, peaks seen between loci at distances <2Mb are often enriched for binding of CTCF and cohesin [41]. In the context of loop extrusion, such peaks can emerge by the reproducible positioning of extruded cohesin loops at CTCF barriers [27]. Peaks in mammalian interphase maps can also emerge due to other complexes, like polycomb [42,43]. Peaks have also been observed and quantified in other contexts, including yeast meiosis and mitosis [20,44,45].

**Fig 6:**
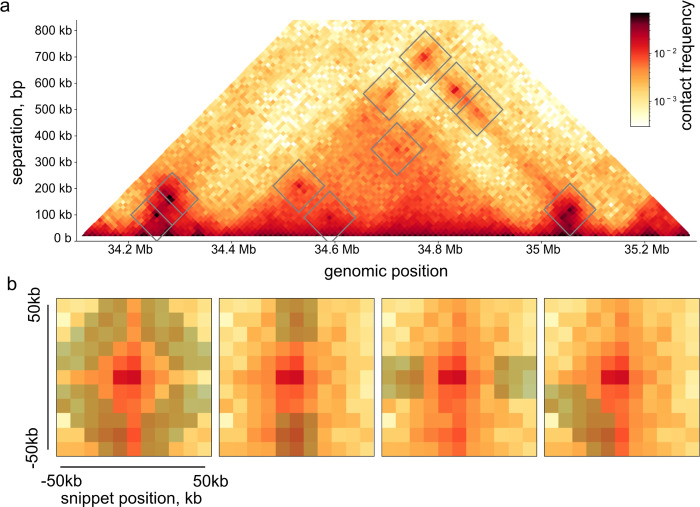
Dots. ***a***. Dots calls from a region of chromosome 17, highlighted by squares on the upper triangular portion of the map. Squares show the size of the region scanned by convolutional kernels. ***b***. illustration of convolutional kernels used for dot calling around one example, from left to right: ‘donut’, ‘top’, ‘bottom’, ‘lowerleft’. Local enrichment at the center pixel is calculated relative to the shaded regions in each kernel.

*Cooltools* implements a HiCCUPs-style [41] approach for peak detection (**Fig 6**): briefly, (i) maps are queried with a dense set of tiles that fit easily into memory, (ii) convolutional kernels are swept across map tiles to score all pixels for local enrichment (iii) significant pixels are determined by thresholding relative to their local background expected values, using a modified Benjamini-Hochberg FDR procedure (iv) adjacent significant pixels are optionally clustered and further filtered by enrichment. *Cooltools* enables control over dot calling at multiple levels, including: the area of the map queried by tiling, allowing user-specified kernels, choosing the FDR, and whether to perform the final clustering and filtering.

The *cooltools* dots module has been used in multiple settings, including: calling peaks in MicroC data [25], calling peaks across conditions [34], and in yeast meiosis [20]. In yeast meiosis, dot-calling benefited from the flexibility in *cooltools* to choose a smaller kernel and relaxed filters in the clustering step.

### Pileups and average snippets

Different classes of genomic elements display distinct local contact patterns depending on their propensity to form features like boundaries or dots. These patterns are typically quantified by extracting local 2D contact maps, or *snippets*, and then averaging snippets to create *pileups* or *average Hi-C maps*. Averaging can reveal genome-wide trends that might otherwise be masked by locus-specific structures or noise in individual snippets. Due to the symmetry of Hi-C maps, pileups come in two varieties: (i) on-diagonal pileups around a set of anchor positions, and (ii) off-diagonal pileups around a set of paired-anchors. On-diagonal pileups are useful for quantifying the strength of boundaries, which define a set of anchors, and off-diagonal pileups are useful for quantifying the strength of dots, which define a set of paired-anchors. Both types of pileup can be useful for determining the relationship between other genomic data (e.g., CTCF binding) with genome folding.

**Fig 7:**
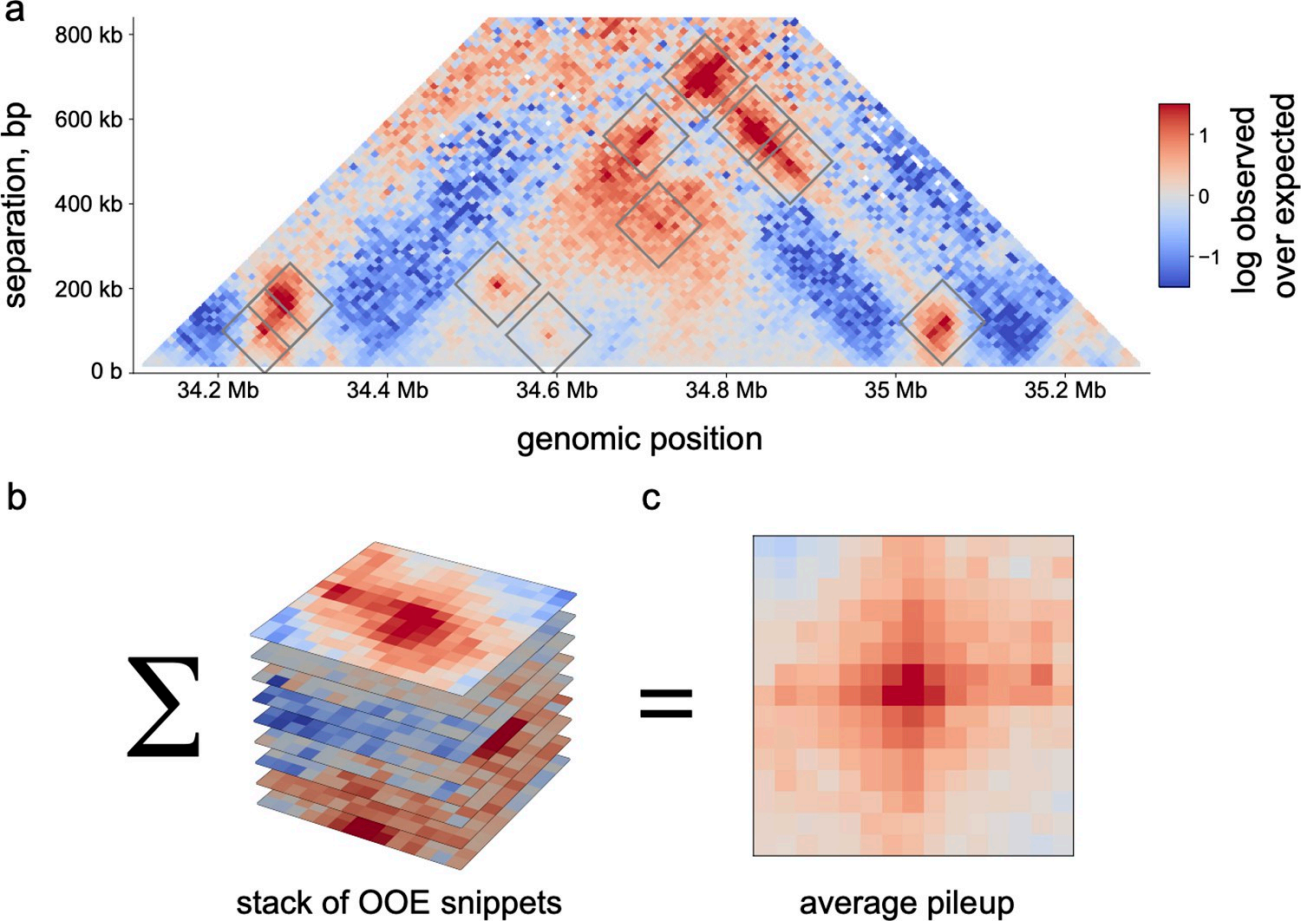
Pileups and average snippets. ***a***. snippets, or regions around called dots, are extracted from the genome-wide map. ***b***. set of extracted snippets. ***c***. average pileup for dots created by averaging the set of snippets.

To calculate pileups (**Fig 7**), *cooltools* first retrieves snippets for the specified set of anchors, a procedure termed *snipping*. Pileups can be built either directly from observed contact map snippets or from snippets normalized by a specified expected table. This is especially useful when the pileups are created for regions with different *P(s)*, and specific expected tables are used for different regions. Users can also specify their choice of balancing weights or whether to calculate pileups directly on raw data. Multiprocessing can be specified to speed up snipping for large sets of anchors.

The modular *cooltools* API facilities interactive analysis of snippets. The flexibility of using raw data for *cooltools* snipping has enabled its use with single-cell Hi-C [35]. The flexibility offered by *cooltools* enables more complex analyses, including considering aggregate-peak-analysis (APA) as a function of genomic distance between anchors [20]. In [25], minimal modifications to the *cooltools* code enabled nucleotide-resolution pileups, revealing patterns of nucleosomes around dots, CTCF anchors and gene starts. Leveraging the flexibility of *cooltools*, *coolpuppy [46]* enables distance-stratified analyses [42] as well as additional functionality for pileups. The separation of feature extraction and quantification has also enabled the quantification of features like TAD strength and peak strength in more sparsely sequenced datasets relative to a more deeply sequenced reference (e.g., for CTCF mutants [47]).

### Coverage (cis/total), smoothing, and sampling

In addition to the main modules described above, *cooltools* provides additional tools for computing coverage, adaptive smoothing, and resampling.

An early observation from Hi-C maps was that contacts are more frequent within chromosomes than between them, even at large genomic separations [48]. This corresponds to the established concept of chromosome territoriality (for review [49]). In *cooltools*, cis and total coverage profiles are calculated by iterating over chunks of pixels followed by summation. One usage of this feature was to determine that dot anchors in yeast meiotic Hi-C maps had higher proportions of cis contacts [20].

Since Hi-C maps are often sparse, especially at high resolutions, understanding and mitigating the impact of sampling noise is often a key component of computational analysis. To aid such analyses, *cooltools* provides a module for generating randomly downsampled coolers. This allowed controlled comparisons across stages of Drosophila germline cell differentiation [50]. To mitigate sampling noise, *cooltools* provides an adaptive smoothing method that iteratively splits observed counts across multiple bins until reaching a user-defined threshold, similar to the approach in Serpentine [51]. Adaptive smoothing has proven useful for applications ranging from visualization to preprocessing data for neural network training [52].

### Availability and future directions

In summary, *cooltools* provides a suite of tools for genome folding analysis that are seamlessly integrated with *cooler*. Available on GitHub since 2017, *cooltools* modernize, extend, and replace the analysis routines in *hiclib [30]*, the first open-source package for processing and analyzing Hi-C data. Currently, *cooltools* and *FAN-C* are the only available Hi-C analysis suites that provide a Python API and operate on the.cool data format (**Table 1**). Benchmarked on Hi-C data, *cooltools* shows better performance, especially at higher resolutions (**S1 Fig**).

The methods in *cooltools* are modular, enabling the development of new analyses. The Python API in *cooltools* yields interpretable and structured outputs (*Pandas* dataframes or *NumPy* arrays), facilitating both data exploration as well as future tool development. For example, since the output of *cooltools* insulation is a *pandas* DataFrame of genomic intervals with additional score columns, the set of boundaries set can be interactively intersected with other genomic data (e.g., CTCF occupancy or motifs) using *bioframe* [15].

The flexibility and breadth of the library make *cooltools* useful across a broad range of contexts. *Cooltools* have been used to analyze Hi-C data across cell states, including mitosis and meiosis, and for organisms ranging from bacteria to yeast to invertebrates to mammals. Analysis of data for non-standard genomes is facilitated by the use of genomic views, which support flexible ordering and naming of chromosomes, as implemented in *bioframe* [15]. Indeed, the flexibility of *cooltools* made it the tool-of-choice for the largest evaluation of experimental Hi-C protocols to date [34].

*Cooltools* provides a foundation for multiple future directions. First, integration with *dask* [53] could be used to further improve the performance of snippers and other tools. Second, *cooltools* could be extended to support emerging types of contact data, including asymmetric contacts (e.g., RNA-vs-DNA [54,55]), and single-cell data [56]. Finally, *cooltools* can be used to develop reproducible analyses for Hi-C datasets. This includes reports, either on single datasets or for cross-dataset analysis, workflow routines for reproducible analysis (e.g., https://github.com/open2c/quaich), and integration with visualization platforms (e.g., HiGlass [57]).

## Supporting information

S1 Fig Performance benchmark of Python API software tool suites. We benchmarked *cooltools and FAN-C* [13] using HFF Micro-C from Krietenstein et al. [25] for two chromosomes, chr2 and chr17. Together, these chromosomes constitute ~325.5 Mb (~10.5% of the human genome). Benchmarks were performed on a per-tool basis at resolutions typically used in published Hi-C literature. Expected was assessed for 1kb, 10kb, 100kb, and 1Mb. Insulation was assessed on 1kb and 10kb maps with window sizes 20 times larger than the resolution (20kb and 200 kb, respectively). Pileups were assessed at resolutions of 1kb, 10kb, and 100kb at window sizes 20 times larger than the resolution (20kb, 200kb, and 2Mb, respectively). At 1kb resolution, FAN-C could not be benchmarked, due to high memory consumption (>94Gb) and running time exceeding 20 mins. Compartments and saddles were calculated in cis at 100kb and 1Mb. All benchmarks are posted on https://github.com/open2c/open2c_vignettes/tree/main/cooltools_manuscript. Benchmarks used cooltools v.0.6.1, FAN-C v.0.9.27, system Linux-6.2.0–37 with 24 CPUs at 3200 GHz (max 4717 GHz). ***a*.** CPU performance benchmark, values above the bars indicate time in seconds. ***b*.** Memory requirements benchmark, values above the bars indicate maximum used memory in MB.
(DOCX)
