# Supplementary material for: Cooltools: Enabling high-resolution Hi-C analysis in Python
Source: PLoS Comput Biol. 2024 May 6;20(5):e1012067. doi: 10.1371/journal.pcbi.1012067 (PMC11098495; doi:10.1371/journal.pcbi.1012067)
Supplement: S1 Fig — We benchmarked cooltools and FAN-C [13] using HFF Micro-C from Krietenstein et al. [25] for two chromosomes, chr2 and chr17. Together, these chromosomes constitute ~325.5 Mb (~10.5% of the human genome). Benchmarks were performed on a per-tool basis at resolutions typically used in published Hi-C literature. Expected was assessed for 1kb, 10kb, 100kb, and 1Mb. Insulation was assessed on 1kb and 10kb maps with window sizes 20 times larger than the resolution (20kb and 200 kb, respectively). Pileups were assessed at resolutions of 1kb, 10kb, and 100kb at window sizes 20 times larger than the resolution (20kb, 200kb, and 2Mb, respectively). At 1kb resolution, FAN-C could not be benchmarked, due to high memory consumption (>94Gb) and running time exceeding 20 mins. Compartments and saddles were calculated in cis at 100kb and 1Mb. All benchmarks are posted on https://github.com/open2c/open2c_vignettes/tree/main/cooltools_manuscript. Benchmarks used cooltools v.0.6.1, FAN-C v.0.9.27, system Linux-6.2.0–37 with 24 CPUs at 3200 GHz (max 4717 GHz). a. CPU performance benchmark, values above the bars indicate time in seconds. b. Memory requirements benchmark, values above the bars indicate maximum used memory in MB. (DOCX) [file pcbi.1012067.s001.docx]

*Supporting information for*

*Cooltools*: enabling high-resolution Hi-C analysis in Python


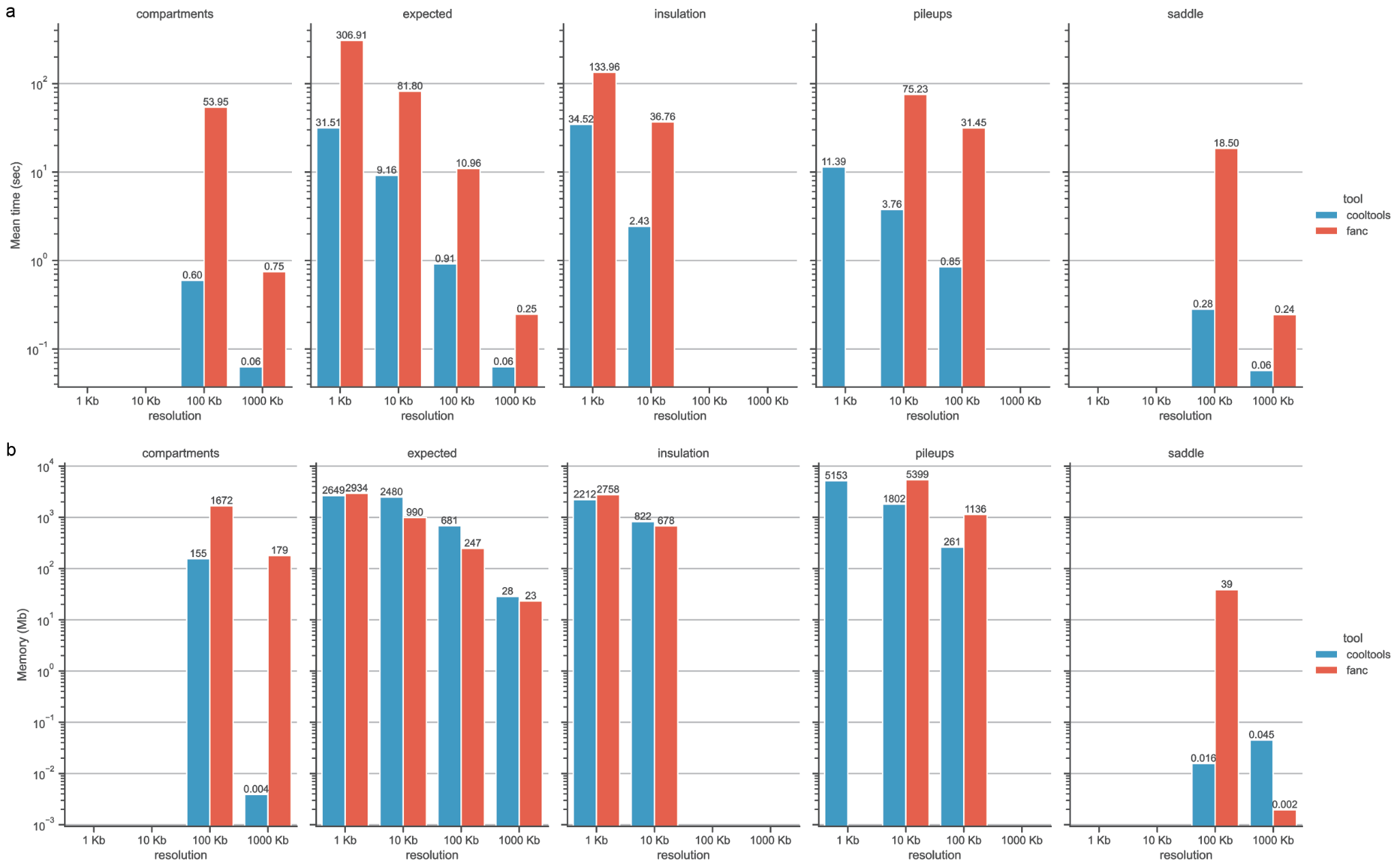


***Supplementary Figure 1. Performance benchmark of Python API software tool suites.***
